# Characterization of *Aspergillus oryzae* mutant and its application in heterologous lipase expression

**DOI:** 10.1016/j.synbio.2024.12.003

**Published:** 2024-12-20

**Authors:** Qinghua Li, Chen Zhang, Jianghua Li, Guocheng Du, Zhaofeng Li, Jingwen Zhou, Guoqiang Zhang

**Affiliations:** a Science Center for Future Foods, Jiangnan University, Wuxi, 214122, China; b School of Food Science and Technology, Jiangnan University, Wuxi, 214122, China; c School of Biotechnology and Key Laboratory of Industrial Biotechnology of Ministry of Education, Jiangnan University, Wuxi, 214122, China; d Jiangsu Province Basic Research Center for Synthetic Biology, Jiangnan University, Wuxi, 214122, China

**Keywords:** Lipase, *Aspergillus oryzae*, Heterologous secretory expression, Multi-copy integration, Transcriptomics

## Abstract

The *Aspergillus oryzae* expression system has been developed into a chassis for the production of heterologous lipases, attributed to its strong capabilities in protein production and secretion, robust post-translational modifications, and favourable safety profile. However, the system's relatively low expression levels remain a challenge, hindering its ability to meet the increasing demands of large-scale production. Strain C19, screened by high-throughput methods combining droplet microfluidics and flow cytometry, was demonstrated to be a potential chassis cell based on fermentation kinetic analysis and transcriptome sequencing. By leveraging the endogenous α-amylase's expression elements and integration sites, a combination of random and site-directed integration strategies was employed to enhance the expression of heterologous lipases in strain C19. As a result, lipase production in shake-flask fermentation reached a titer of 113.6 U/L. The study further demonstrated that the different α-amylase gene loci could serve as effective integration sites for the multi-copy expression of heterologous proteins because the lipase activity of the 3-amylase site integrated strain C19#1-ABC was 3.3 times higher than that of C19#1. Furthermore, fermentation results in a 5-L bioreactor indicated that optimization of fermentation processes and facilities had the potential to further increase heterologous protein expression levels. These findings offered valuable insights into the advancement of *A. oryzae* expression systems and the potential for scaling engineered strains for industrial applications.

## 1 Introduction

*Aspergillus oryzae* is a filamentous fungus that has been granted Generally Recognized as Safe (GRAS) status by the U.S. Food and Drug Administration (FDA) [1]. Its robust capabilities for protein expression, secretion, and post-translational modification make it a promising candidate for development as an excellent chassis cell [2]. This potential has been increasingly realized in the expression and production of organic acids, industrial enzymes, and secondary metabolites [3]. Despite *A. oryzae*'s strong ability to synthesize and secrete proteins, the system still encounters challenges, particularly the low expression levels of heterologous proteins [4,5]. The expression levels of non-fungal proteins are typically one to two orders of magnitude lower than those of homologous proteins, which is a pervasive and urgent issue in the *Aspergillus* expression system [6].

Several strategies have been employed to address this challenge, including the use of strong constitutive or inducible promoters, the establishment of efficient secretion signals, and the construction of protease-deficient strains, all of which have yielded promising results [7]. Additionally, integrating multiple gene copies within *Aspergillus* has been shown to enhance the expression levels of endogenous proteins [8]. Consequently, under the selection of appropriate integration sites, the integration of multiple copies of heterologous genes is expected to similarly improve the expression levels of the corresponding proteins [9]. However, the low efficiency of genetic manipulation and expression optimization in *A. oryzae* has limited the development and application of the *A. oryzae* expression system [10]. In recent years, technological advancements have led to the establishment and optimization of high-throughput screening techniques and efficient genome editing tools for *A. oryzae* [2,[11], [12], [13]]. These innovations have laid a solid foundation for enhancing the transformation, screening, optimization, and application of *A. oryzae* expression systems.

Lipase (EC 3.1.1.3), commonly referred to as triacylglycerol lipase, is a naturally occurring enzyme renowned for its capacity to hydrolyze fats and lipids [14]. Owing to their versatility and distinct characteristics, such as high efficiency, precise control, and environmental friendliness, lipases are widely applicable across various fields, including medicine, biotechnology, and the food industry [15]. To meet the demands of industrial production, researchers have undertaken systematic and efficient screening to identify and explore novel lipase-producing strains [16]. However, a common challenge associated with wild-type strains is their typically low expression levels [17]. Consequently, achieving high-level heterologous expression of lipases is a key objective for fully harnessing the potential of these enzymes.

In this study, the mutant strain C19 was selected as the candidate for achieving efficient lipase expression in *A*. *oryzae*. C19 demonstrated high endogenous α-amylase production, and transcriptome analysis further underscored its potential as a robust platform for developing an efficient *A. oryzae* expression system for heterologous protein production. Initially, lipase expression and secretion were accomplished by screening optimal integration sites through random integration. Subsequently, the lipase gene was specifically integrated into the endogenous amylase gene locus of *A. oryzae* using the clustered regularly interspaced short palindromic repeats (CRISPR) and CRISPR-associated protein 9 (CRISPR/Cas9) system, resulting in a significant enhancement of expression levels. The high-expression lipase strains were then evaluated in a bioreactor, providing valuable insights into the efficient expression of heterologous proteins and process scale-up in the *A. oryzae* expression system.

## 2 Materials and methods

### 2.1 Strains, media, and culture conditions

In this study, *A. oryzae* RIB40 (ATCC42149) and the high-yield α-amylase-producing strain C19 were used as host cells [13]. *Escherichia coli* JM109 was used for the construction of CRISPR/Cas9-related module and edit plasmids. The *A. oryzae* spores were activated by culturing on potato dextrose agar (PDA) medium at 30 °C for 3–5 days. After maturation, the spores were harvested by washing and filtering with deionized water, resulting in a spore suspension. The spore suspension was then inoculated into adjusted Czapek Dox (ACD) liquid medium (containing 2 % glucose, 0.3 % NaNO_3_, 0.1 % K_2_HPO_4_, 0.05 % KCl, 0.05 % MgSO_4_·7H_2_O, and 0.001 % FeSO_4_·7H_2_O) and incubated at 30 °C with shaking for 20 h to obtain fresh mycelia for protoplast preparation. Positive transformants during the transformation process were selected using high-osmolarity ACD medium (0.8 M NaCl added) supplemented with either 200 μg/mL of hygromycin or 0.5 μg/mL of pyrithiamine for selection. The culture medium used for *A. oryzae* α-amylase or lipase fermentation contained the following ingredients: 2 % dextrin, 0.5 % peptone, 0.1 % yeast extract, 0.1 % NaNO_3_, 0.05 % KH_2_PO_4_, 0.05 % MgSO_4_·7H_2_O, and 0.001 % FeSO_4_·7H_2_O. Common chemical reagents were purchased from the Sinopharm (Shanghai, China).

### 2.2 Plasmid construction and molecular manipulation

Based on the plasmid pC9sgR-Model, the protospacer sequence was replaced by PCR mutation to obtain the pC9sgR-yA-amyABC module plasmid, and then the Cas9-sgRNA-yA-amyABC module fragment was obtained through PCR. The fragments were assembled with the linearized pPTR II vector using GeneArt™ Gibson Assembly® HiFi Cloning Kits (Thermo Fisher Scientific, Shanghai, China) to obtain editing plasmids. Other plasmids were constructed using conventional PCR and Gibson assembly, and the plasmids and functions used in this study are shown in Table 1. The main primers used in this study are listed in Table 2.

**Table 1:** The main plasmids used in this study.

Plasmid	Function	Source
pPTR II	Vector for editing plasmids	Takara (Code No. 3622)
pC9sgR-Model	Plasmid for protospacer change	This study constructed
pC9sgR-yA-amyABC	Module plasmid targeting gene *yA* and three amylase genes	This study constructed
pAmy-TLL-Ble	TLL integrated module with bleomycin resistance	This study constructed
pAmy-TLL-ptrA	TLL integrated module with pyrithiamine resistance	This study constructed
pTR-C9sgR-yA-amyABC	Editing plasmid targeting gene *yA* and three amylase genes	This study constructed

**Table 2:** The main primers used in this study.

Primer	Sequence (5′-3′)
Assemble-F	TGTGGATAACCGTATTACCGCCTTTGAGTGAGCTGATTTAATTAAGCCGACATAGCTGTTTCCGCTGAGG
Assemble-R	GCTACAGGGCGCGTACTATGGTTGCTTTGACGTATGCGTCCCTGGGAG
Protospacer-amylase-F	GATGACTTGAGTTTTAGAGCTAGAAATAGCAAG
Protospacer-amylase-R	TGCAGTGCCGTGCATCATCCGTGAATCG
Donor-TLL-F	AGCGTTAATTAATCGCTTACATGGGG
Donor-TLL-R	CGGCCGCAAGTTCTAAACTTAGG
Check-TLL-F	AAGGACCACCTCTAGGCATCG
Check-TLL-R	ACCATCATGACCTCTACAACCTGAAC

### 2.3 Preparation and transformation of *A. oryzae* protoplasts

The liquid ACD medium was inoculated with a spore suspension obtained by eluting mature PDA plates. The inoculated medium was incubated with agitation at 30 °C for 16 h, yielding fresh *A. oryzae* hyphae after subsequent filtration and washing steps. The mycelium was then mixed with the enzyme solution in the optimized proportions and incubated at 30 °C for 1–2 h [18]. The resulting protoplasts were collected and resuspended in STC buffer (containing 1.2 M Sorbitol, 50 mM CaCl_2_, and 50 mM Tris-HCl; pH 7.5). For the transformation process, 10 μg of DNA was added to 200 μL of protoplasts. Simultaneously, 80 μL of PEG4000 (with a weight/volume ratio of 40 %) was introduced, and the mixture was gently combined and kept on ice for 30 min. Subsequently, 1.5 mL of PEG4000 was added to the mixture, followed by another 30-min incubation at room temperature. Finally, the transformation solution was evenly spread onto the corresponding plates.

### 2.4 *A. oryzae* colony PCR

A small amount of fresh mycelia was selected and placed in lysis buffer (containing 0.5 % Triton X-100, 1 mM EDTA, and 50 mM NaOH). Then, the mixture was incubated at 95 °C in a PCR machine for 20 min [18]. After centrifugation, the supernatant obtained can be used as a template for the standard PCR system.

### 2.5 *A. oryzae* growth curve determination

Inoculate fresh spore suspensions into an ACD culture medium and cultivate it at 30 °C with shaking. Every 12 h, three shaking flasks were removed, and all mycelia were collected f and dried [19]. Finally, the dried mycelia were weighed to calculate the mycelial dry weight.

### 2.6 Shake flask fermentation

After elution and filtration from the plate, the concentration of the spore suspension was adjusted to 10^7^ CFU mL^−1^ by using a hemocytometer. Then, the spore suspension was inoculated at a rate of 3 % into the fermentation medium (60 mL in a 250 mL flask) and shaken at 30 °C for 72 h. During this period, the shaking speed was set to 180 rpm for the first 24 h and increased to 220 rpm after 24 h to minimize the impact of wall growth on the fermentation process.

### 2.7 α-Amylase enzyme activity assay

The 3,5-dinitrosalicylic acid (DNS) method was used to determine the enzymatic activity of α-amylase in the fermentation broth supernatant [20]. The specific steps were as follows: A 2 % starch substrate solution was prepared in 10 mM pH 5.6 citrate buffer. Then, 900 μL of the preheated substrate solution was reacted with 100 μL of an appropriately diluted enzyme solution at 50 °C for 10 min, and then 2 mL of DNS reagent was added to terminate the reaction. The colour was developed in a boiling water bath for 10 min, cooled and diluted to 25 mL with deionized water, and the absorbance was measured at 520 nm. The inactivated enzyme was used as a control. According to the standard curve prepared from glucose, the content of reducing sugar (in glucose units) in the system and the α-amylase activity in the fermentation broth supernatant were calculated. Enzyme activity was defined as follows: under certain conditions (50 °C, pH 5.6), the amount of enzyme that produces 1 mg of glucose after 1 min reaction was one unit of enzyme activity.

### 2.8 Lipase enzyme activity assay

Enzyme activity is defined as the quantity of enzyme necessary to catalyze the conversion of *p*-nitrophenol palmitate into 1 μmol of *p*-nitrophenol per unit of time under specified conditions. The procedure to assess enzyme activity involves combining 1.8 mL of a 0.3 % (w/v) *p*-nitrophenol palmitate (*p*-NPP) solution with 200 μL of the enzyme solution. The enzymatic reaction is conducted at a controlled temperature of 40 °C for a defined period of 10 min. Following this incubation, 1 mL of 95 % ethanol was added to terminate the reaction. The solution was then subjected to centrifugation, and the absorbance of the resulting reaction mixture was measured at a wavelength of 410 nm [21].

### 2.9 5-Liter bioreactor fermentation

The mature PDA plate was eluted and filtered with deionized water to obtain a spore suspension, and the concentration was adjusted to 10^7^ CFU mL^−1^. A fermentation tank with a liquid volume of 2.5 L was inoculated at 5 %, and batch fermentation was conducted at 1.0 VVM and 400 rpm. Sampling was performed every 12 h to detect the biomass and fermentation enzyme activity.

### 2.10 Transcriptome analysis

Fresh mycelia from high-producing α-amylase strain C19 and wild-type strain RIB were collected after culturing in a fermentation medium for 24 h. Library construction and RNA sequencing were performed by GENE DENOVO (Guangzhou, China). Total RNA was extracted by the Trizol method and used for library construction.

## 3 Results and discussions

### 3.1 Strain C19: a potential *A*. *oryzae* chassis cell

**Fig. 1:**
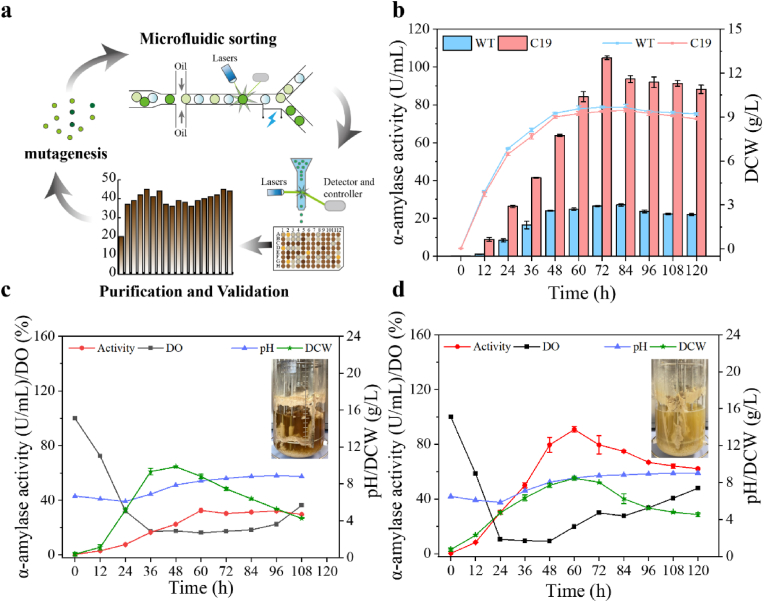
Evaluation of growth and fermentation kinetics of high-yielding strain C19. (**a**) Flowchart for high-throughput screening of *A*. *oryzae*; (**b**) Comparison of fermentation kinetics between C19 and wild-type in shake flasks; (**c**) Fermentation kinetics of wild-type in bioreactors; (**d**) Fermentation kinetics of C19 in bioreactors.

To efficiently obtain superior *A*. *oryzae* mutant strains, we have previously developed a high-throughput screening platform by integrating droplet microfluidics and flow cytometry (Fig. 1a) [13]. This platform can complete the screening and verification of 250,000 mutants within 10 days with high accuracy. Using this platform, the high α-amylase-producing C19 strain was efficiently screened and demonstrated strong genetic stability in subsequent passaging experiments. The production performance of the C19 strain was evaluated in both shake flasks and bioreactors. In shake-flask cultures, C19 exhibited hyphal growth comparable to that of the wild-type strain, while its α-amylase expression levels were significantly higher (Fig. 1b), indicating enhanced protein production capacity. In bioreactors, although C19 exhibited higher α-amylase activity than the wild-type, its expression levels were lower than those observed in shake-flask cultures. Furthermore, C19 showed reduced growth rates and biomass in bioreactors, despite its faster initial oxygen consumption rate (Fig. 1c and d). These results were inconsistent with expectations, given the richer nutrient conditions in the bioreactor, suggesting that hyphal growth may have been inhibited. After fermentation, the bioreactor was left to stand for 1 h. As the mycelial balls settled, the fermentation broth of the wild-type strain became clear, whereas that of C19 remained turbid because of the presence of numerous mycelial fragments. This suggests that the shear forces generated by the stirring paddles caused greater damage to C19, leading to hyphal fragmentation, which in turn reduced its growth rate and biomass. Despite these unfavourable conditions, C19 maintained relatively high levels of α-amylase expression, highlighting its potential as a robust protein expression system.

### 3.2 Transcriptomic analysis of strain C19

**Fig. 2:**
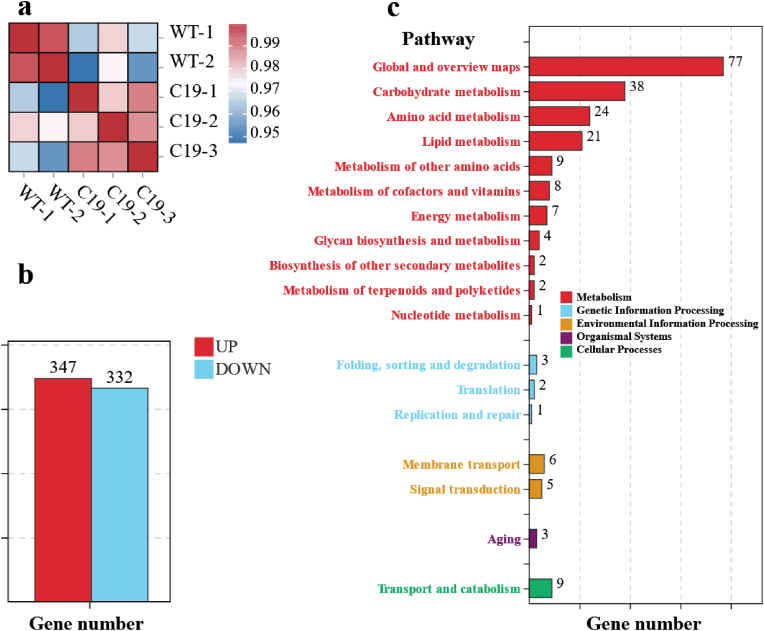
Transcriptional analysis of strain C19. (**a**) Pearson's correlation analysis of samples based on the TPM values; (**b**) Significantly differentially expressed genes (log2Fold change ≥2, Pvalue ≤0.05) in strain C19 compared with the reference strain RIB40; (**c**) Statistical information on the number of genes in major differential metabolic pathways.

To investigate the high-yield α-amylase mechanism of the C19 strain, transcriptomic analysis was performed (Fig. 2). Pearson's correlation analysis of the samples, based on Transcripts Per Million (TPM) values, demonstrated good reproducibility among the biological replicates (Fig. 2a). Compared with the wild-type strain, 679 genes showed significant differences in expression levels (log2Fold change ≥2, P-value ≤0.05) (Fig. 2b). Of these genes, 347 were upregulated, and 332 were downregulated. The Kyoto Encyclopedia of Genes and Genomes (KEGG) analysis revealed that these genes are involved in several key metabolic pathways, including carbohydrate, amino acid, lipid, and other acid metabolisms. They are also linked to processes such as transport and catabolism, cofactor and vitamin metabolism, energy metabolism, membrane transport, and signal transduction (Fig. 2c). These findings suggest that the expression differences in the C19 strain primarily occur in intracellular processes such as carbon metabolism, amino acid metabolism, energy metabolism, and membrane transport, all of which are closely related to the strain's ability to efficiently express proteins. In addition, changes in the expression of genes related to glycan biosynthesis and metabolism can affect the biosynthesis and integrity of the cell wall, thereby influencing the sensitivity of C19 hyphae to shear forces. Further research on the relevant genes and regulatory mechanisms, based on the obtained transcriptional data, would be conducted to develop a highly efficient heterologous protein expression system for *A*. *oryzae*.

### 3.3 Expression of heterologous lipase in *A. oryzae* via random integration

**Fig. 3:**
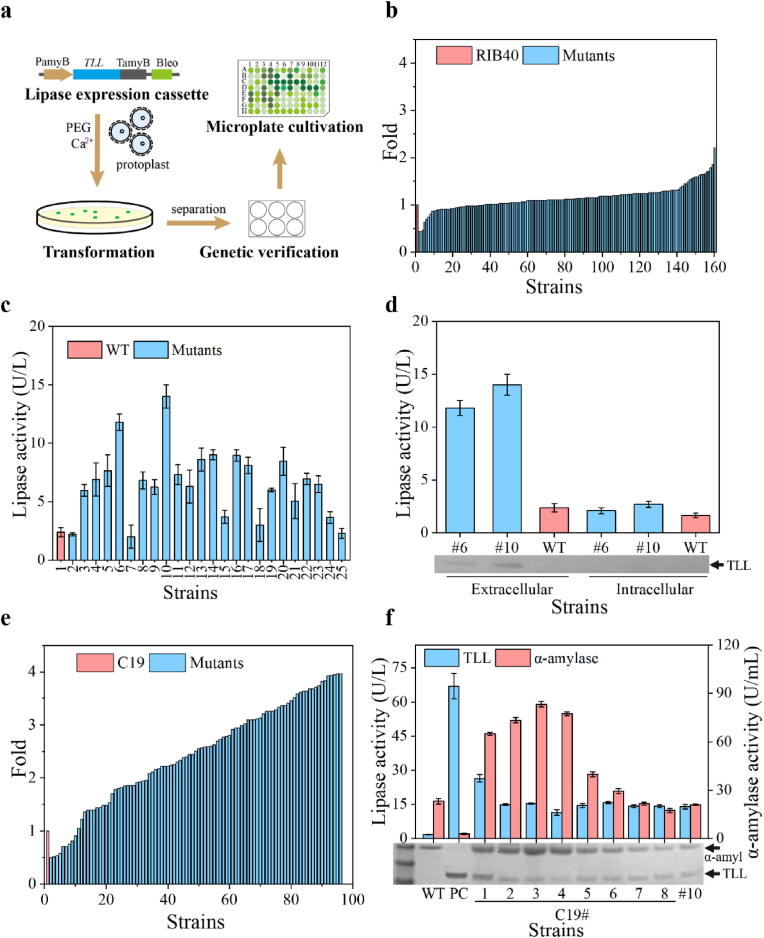
Integrated expression of heterologous lipase in RIB40 and C19 (**a**) Flow chart of construction and random integration of heterologous lipase expression cassette; (**b**) Microplate culture and screening of lipase expressing strains based on RIB40; (**c**) Verification of lipase expression strains derived from RIB40; (**d**) Extracellular and intracellular expression of lipase expression strain #6 and #10; (**e**) Microplate culture and screening of lipase expressing strains based on C19. (**f**) Verification and screening of high-producing lipase strains derived from C19; PC is strain *A. oryzae*-TLLs (*amyB::TLL*, *amyC::TLL*).

The *A*. *oryzae* expression system is highly valued for its strong expression and secretion capabilities, as well as its precise post-translational modifications. However, the significant disparity in expression levels between homologous and heterologous proteins remains a notable challenge. The enhanced expression of endogenous α-amylase in the C19 strain highlights its potential as an excellent chassis cell. To further assess its ability to express heterologous proteins, the lipase gene *TLL* from *Thermomyces lanuginosus* was selected for comparative expression experiments. Lipases typically possess complex three-dimensional structures and often require proper folding and post-translational modifications within cellular organelles, such as the endoplasmic reticulum and Golgi apparatus to function catalytically [22]. *TLL* was engineered into an expression cassette utilizing the endogenous *A. oryzae* α-amylase promoter (PamyB), along with its associated signal peptide and terminator (TamyB) (Fig. 3a). PamyB is a robust and widely used promoter in the *Aspergillus* expression system, known for its inducibility by starch or dextrin and its suppression by glucose [23]. The constructed expression module PamyB-TLL-TamyB-Bleo, which included a bleomycin resistance marker, was linearized and randomly integrated into the genome of strain RIB40 and C19. Following integration, a thorough selection process was conducted based on bleomycin resistance. The positive transformants were identified through separation, purification, and verification. Microplate-based cultivation and selection were used for the lipase-producing strains based on enzyme activity (Fig. 3b).

When random integration was performed using RIB40 as the chassis cell, 24 positive strains were identified. These strains were then subjected to shake-flask fermentation to evaluate their lipase production, resulting in 22 strains demonstrating significantly elevated lipase enzyme activity, indicating successful expression and secretion of the heterologous lipase in *A. oryzae* (Fig. 3c). The differences in the lipase expression levels among the positive strains may be due to the influence of random integration sites. Besides, an analysis of the intracellular and extracellular expression of lipase for the relatively high-activity positive strains (#6 and #10) revealed no detectable intracellular enzyme activity (Fig. 3d), indicating that *A. oryzae* is a suitable expression system for lipase expression and the endogenous α-amylase signal peptide of *A. oryzae* effectively facilitates the secretion of heterologous lipase. However, the extracellular lipase enzyme activity remained relatively low, with strains #6 and #10 exhibiting activities of only 11.8 U/L and 14 U/L, respectively (Fig. 3d).

In the random integration experiment using strain C19 as the chassis cell, the top eight strains with the highest expression levels were selected for shake flask rescreening (Fig. 3e). The results showed that the maximum extracellular lipase activity produced by strain C19#1 was 26.5 U/L, which was 0.9 times higher than that of strain #10 (Fig. 3f). This showed that the C19 strain has greater potential than RIB40 in expressing heterologous proteins, and when heterologous genes are induced by promoter PamyB, the selection of *A*. *oryzae* strains with high endogenous α-amylase expression levels is beneficial to improving the expression level. However, the fold increase in the expression level of heterologous lipase compared with that of RIB40 was significantly lower than that of endogenous α-amylase. Although the integration site of the lipase has some influence on expression, it also reflects differences in the expression levels of homologous and heterologous proteins in the *A*. *oryzae* expression system. In addition, since the expression element of lipase is derived from the endogenous α-amylase of *A. oryzae*, there is a certain competitive relationship between the expression of lipase and α-amylase, which explains the decrease in α-amylase expression levels (Fig. 3f). The differences in lipase expression levels between transformants were mainly attributed to differences in integration sites. Finally, the strain C19#1, which displayed relatively high expression levels of both lipase and α-amylase, was selected for further experiments. It should be noted that the positive control, *A. oryzae*-TLLs (*amyB::TLL*, *amyC::TLL*), was obtained in a previous study [18].

### 3.4 Multicopy integration improves the secretory expression of heterologous lipase

In general, the integration of multiple gene copies in *Aspergillus* can enhance the expression levels of homologous proteins [24]. Similarly, the introduction of additional exogenous gene copies can increase protein production to a certain extent. The α-amylase gene in *A. oryzae* comprises three copies (*amyA*, *amyB*, and *amyC*), located on chromosomes 2, 5, and 3, respectively [25]. Sequence alignment revealed that the gene expression cassettes of *amyB* and *amyC* are identical, whereas the coding DNA sequence of *amyA* contains two base mutations, and the terminator of *amyA* was also different. Therefore, the designed CRISPR/Cas9 simultaneously targeted all three genes.

**Fig. 4:**
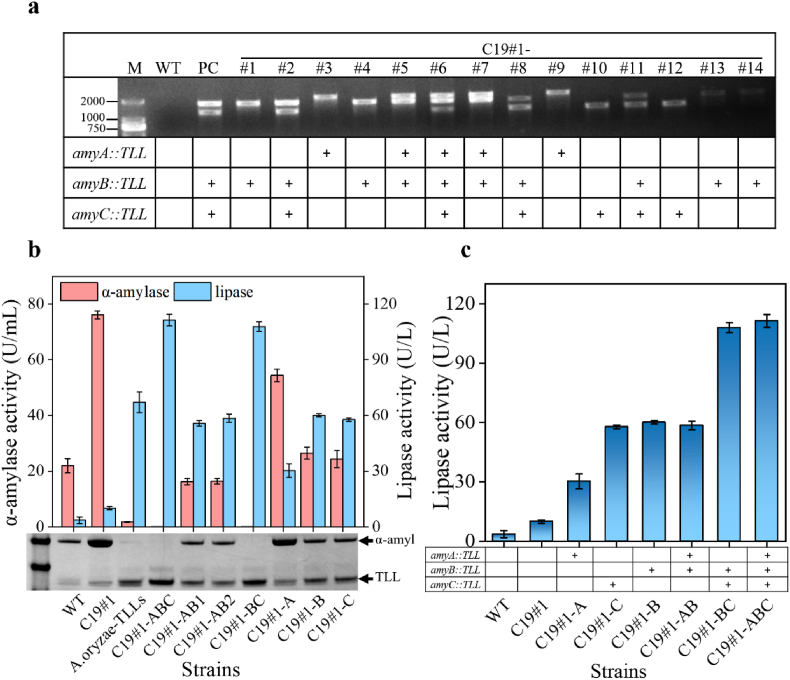
Integrated expression of lipase gene at α-amylase site of *A. oryzae*. (**a**) Site-specific integration of lipase genes in the *A. oryzae* genome; (**b**) Enzyme activities of lipase and α-amylase in different expression strains; (**c**) The comparison of expression strength at different α-amylase integration sites in *A. oryzae*.

To ensure that the integration module PamyB-TLL-TamyB could undergo homologous recombination at the three target sites, the C-terminal homologous arms retained a portion of the coding DNA sequence from α-amylase. Moreover, the integration site selection based on amylase was favourable for eliminating competition between lipase and amylase expression. Utilizing the *yA* morphological gene as a selection marker, 14 positive transformants were chosen for colony PCR verification. The results indicated that all 14 positive transformants successfully integrated the lipase gene, with transformant #6 successfully integrating three copies, five transformants integrating two copies, and eight transformants integrating one copy (Fig. 4a). These strains encompassed six out of seven possible combinations of lipase gene integration except for the double-replacement strain of *amyA* and *amyC*. Seven selected strains (#1, #3, #5, #6, #7, #8, and #10) were evaluated through shake flask fermentation (Fig. 4b). To more clearly indicate the lipase integration sites, they were renamed as C19#1-XXX. For instance, the integration site of C19#1-#1 is *amyB*; therefore it was renamed C19#1-B. The results demonstrated that C19#1-ABC exhibited the highest lipase activity at 113.6 U/L and completely lost extracellular amylase activity, indicating that the integration of multiple copies of exogenous genes is indeed advantageous for increasing expression levels. Additionally, compared with C19#1, the lipase expression level of C19#1-ABC increased by 3.3 times, which meant the amylase gene sites were favourable for the expression of heterologous proteins in *A. oryzae*. Furthermore, the C19#1-BC strain, which replaced *amyB* and *amyC*, exhibited lipase activity (108 U/L) similar to that of C19#1-ABC, consistent with the lower expression of *amyA* in *A. oryzae*. Comparisons among C19#1-A, C19#1-B, and C19#1-C also yielded similar results (Fig. 4b).

To better evaluate the influence of different α-amylase integration sites on the expression of heterologous genes, the strains were sorted based on integration sites and the number of integrations (Fig. 4c). The results indicated that the strength of heterologous lipase expression at the three α-amylase integration sites in the order from low to high was *amyA* < *amyC* < *amyB*, consistent with the expression levels of the three α-amylase genes in *A*. *oryzae* [26]. The difference in expression levels between *amyC* and *amyB* was minimal, with *amyA* exhibiting a lower expression. Consequently, the strength of double gene expression, from low to high, was *amyAC* < *amyAB* < *amyBC*. Although lipase-expressing strain that integrated *amyAC* was not obtained, based on the situation of *amyB* and *amyAB*, it can be inferred that the difference in expression levels between *amyC* and *amyAC* is minimal. Moreover, the expression levels of *amyABC* and *amyBC* did not differ significantly.

### 3.5 Fermentation of lipase expression strains in 5-L bioreactor

**Fig. 5:**
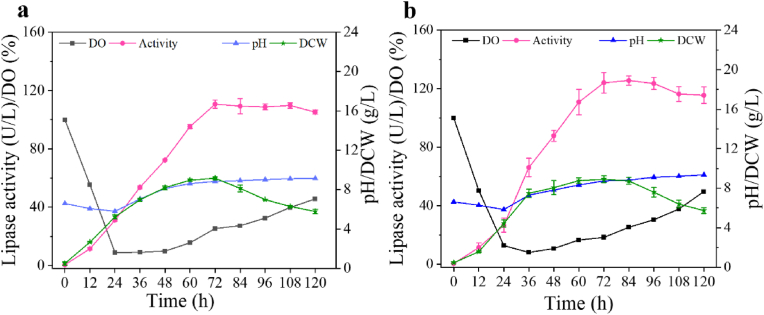
Comparison of the fermentation of different strains in a 5-L bioreactor. (**a**) The fermentation of the strain C19#1-BC; (**b**) The fermentation of the strain C19#1-ABC.

To further assess the lipase production performance of strains C19#1-BC and C19#1-ABC, the fermentation in a 5-L bioreactor was compared (Fig. 5). The results revealed that the oxygen consumption rates of strains C19#1-BC and C19#1-ABC were comparable to that of the parent strain C19. However, these strains exhibited significantly reduced growth rates and biomass accumulation. This reduction was mainly attributed to the decreased expression levels of α-amylase, which in turn led to a marked decrease in the dextrin utilization rate by *A*. *oryzae*. Under conditions of nutrient limitation and dextrin induction, the promoter PamyB facilitated a substantial increase in lipase expression. During batch fermentation at 72 h, the lipase activities of strains C19#1-BC and C19#1-ABC were measured at 110.7 U/L and 125.6 U/L, respectively. These results further underscore the limited expression potential of the *amyA* locus in *A. oryzae*. Theoretically, the nutrient environment in a bioreactor is superior to that in shake flasks, and protein expression levels are generally higher in bioreactors. However, the lipase expression level of *A*. *oryzae* during batch fermentation was not significantly different from that during shake-flask fermentation. Wall growth and aggregation were observed in the bioreactor, which affected biomass and protein expression levels in the fermentation broth. Although wall growth can also occur during shake-flask fermentation, the shear force generated by the agitator in the bioreactor breaks up mycelial clumps into dispersed mycelia, leading to increased wall growth and aggregation in the later stages of bioreactor fermentation, which is the main reason for the significant decrease in biomass [27]. Therefore, achieving a more efficient fermentation process and controlling the balance between *A. oryzae* mycelial growth and oxygen levels during fermentation while avoiding abnormal aggregation and wall growth inside the bioreactor is of significant importance for the industrial production of *A. oryzae* [28].

## 4 Conclusion

The *A*. *oryzae* expression system demonstrates a significant potential for the efficient production of heterologous lipases, largely owing to its robust protein expression, secretion capabilities, and sophisticated post-translational modifications. In this study, the successful multi-copy integration and expression of heterologous lipase in *A. oryzae* mutant C19 were achieved through a combination of random and site-specific integration. Although the expression system shows a preference for the source of the protein, the α-amylase gene's integration site has proven to be an effective target for enhancing the expression of heterologous proteins. Therefore, the development of engineered strains with improved α-amylase production, achieved through mutagenesis or genome engineering, coupled with high throughput screening and comprehensive omics analyses, holds promise for advancing *A. oryzae* as a powerful heterologous expression platform.

## CRediT authorship contribution statement

**Qinghua Li:** Writing – original draft, Visualization, Methodology, Investigation, Data curation. **Chen Zhang:** Investigation, Data curation. **Jianghua Li:** Supervision, Conceptualization. **Guocheng Du:** Conceptualization. **Zhaofeng Li:** Conceptualization. **Jingwen Zhou:** Conceptualization, Supervision. **Guoqiang Zhang:** Supervision, Funding acquisition, Conceptualization.

## Data availability statement

The data supporting the findings of this study are available within the paper. The datasets generated and analyzed during the current study are available from the corresponding author upon request.

## Declaration of competing interest

The authors declare that they have no known competing financial interests or personal relationships that could have appeared to influence the work reported in this paper.
